# A Single Arm Pilot Observational Study to Evaluate the Safety and Feasibility of a Pre‐Operative Very Low Calorie Diet in Severely Obese Patients With Endometrial Cancer

**DOI:** 10.1002/cnr2.70172

**Published:** 2025-04-03

**Authors:** Chloe Ayres, Hanna Burbidge, Jayna Garratt, Ganendra Raj Mohan, Yee Leung, Stephanie Jeffares, Sanela Bilic, Paul A. Cohen

**Affiliations:** ^1^ Western Australian Gynaecologic Cancer Service, Department of Obstetrics and Gynaecology, Women and Newborn Health Service, King Edward Memorial Hospital Subiaco Western Australia Australia; ^2^ Department of Nutrition and Dietetics Women and Newborn Health Service, King Edward Memorial Hospital Subiaco Western Australia Australia; ^3^ Department of Gynaecological Oncology St John of God Subiaco Hospital Subiaco Western Australia Australia; ^4^ Division of Obstetrics and Gynaecology Medical School, University of Western Australia Crawley Western Australia Australia

**Keywords:** endometrial cancer, obesity, Prehabilitation, surgery, very low‐calorie diet (VLCD)

## Abstract

**Background:**

Pre‐operative very low calorie diets (VLCDs) can achieve rapid and safe weight loss, yet no studies have evaluated VLCDs in the severely obese endometrial cancer population prior to surgery. Our aim was to evaluate the safety and feasibility of a 4–6 week pre‐operative nutritional intervention with the Optifast VLCD prior to surgery in patients with clinical stage 1, Grade 1 endometrioid endometrial adenocarcinoma and a body mass index ≥ 35 kg/m^2^.

**Methods:**

This was an investigator‐initiated single‐arm prospective observational study. Co‐primary endpoints were safety and feasibility. Secondary endpoints were changes in anthropometric measures, blood pressure, biochemistry, perioperative complications, length of stay and final tumour stage. Tolerability and compliance of the VLCD were assessed by fortnightly questionnaires and urinary ketones.

**Results:**

Twenty‐eight patients were enrolled, of which 25 underwent the intervention. 22/25 patients (88%) completed at least 4 weeks of Optifast. Mean (SD) age was 56.4 (6.3) years, and mean body mass index (BMI) was 45.2 (7.1) kg/m^2^. Significant decreases in weight (mean 8.2 kg [3.6]), BMI (mean 3.1 kg/m^2^ [1.3]), waist and hip circumference (mean 5.7 [6.5] and 4.5 cm [4.1], respectively), and diastolic blood pressure (10 mmHg [14.1]) were observed (*p* < 0.001 for all). One patient had a flare of gout. All patients had laparoscopic surgery without adverse events. Optifast was considered acceptable, and compliance was 40% to 61.9%. Eighty‐eight percent (22/25) of patients had FIGO 2009 Stage 1A Grade 1 endometrioid endometrial adenocarcinoma on final staging.

**Conclusions:**

A 4–6 week pre‐operative VLCD in severely obese clinically low‐risk endometrial cancer patients appears safe, feasible and well tolerated.

## 1 Introduction

Very low‐calorie diets (VLCDs) can provide safe and rapid weight loss in the period immediately preceding surgery. VLCDs have been used successfully to reduce the perceived technical difficulty and perioperative complications in bariatric and general surgery but have not been evaluated in severely obese patients with endometrial cancer prior to surgery [1, 2, 3, 4, 5, 6]. Endometrial cancer (EC) is the most common gynaecologic malignancy in Australia, accounting for an estimated 3267 new cases in 2021 [7]. The incidence of EC has increased by 132% in the past three decades, consistent with a rise in the prevalence of risk factors, particularly obesity and an ageing population [8].

Minimally invasive surgery is the mainstay treatment of early‐stage low‐grade EC, which includes hysterectomy, bilateral salpingo‐oophorectomy, and sentinel lymph node biopsy for surgical staging [9]. Obesity poses significant challenges to the performance of safe surgery regardless of surgical approach. There has been increasing interest in prehabilitation programs, including nutritional interventions, for cancer patients preparing for major surgery [10, 11, 12, 13]. Aggressive yet empathetic attempts should be made in obese women to achieve acceptable weight loss in a short period of time so that surgery can be safely offered without compromising oncologic outcomes. VLCDs, as a total meal replacement, have had limited success when used without medical and dietetic supervision, which has led to scepticism about their effectiveness. However, VLCDs rank second after bariatric surgery in their ability to help overweight or obese adults lose weight [14]. Patients with a Body Mass Index (BMI) ≥ 35 can be expected to lose 1.5–2.5 kg per week [15]. VLCDs safely and effectively achieve significant reductions in body weight, liver volume and visceral and subcutaneous adipose tissue [16]. Many of the metabolic and physiologic effects of VLCDs are also beneficial, such as improvement in insulin sensitivity and fasting plasma glucose levels enabling a reduction or even cessation of diabetic medications, lowering of blood pressure and serum triglyceride values [17, 18]. VLCDs are also conducive to ketosis, which can help suppress hunger and preserve lean muscle tissue [19]. The compliance rate for short‐term VLCDs of 2–24 weeks duration is surprisingly high at 86%–96% [16].

The primary objective of our study was to evaluate the feasibility and acceptability of a 4–6 week pre‐operative nutritional intervention with the VLCD Optifast prior to definitive endometrial cancer surgery in severely obese women with low‐grade, clinically early‐stage EC.

## 2 Methods

This was an investigator‐initiated single‐arm prospective observational pilot study. The study protocol is available in the Data S2. The study was conducted in accordance with The Strengthening the Reporting of Observational Studies in Epidemiology (STROBE) Statement: guidelines for reporting observational studies [20]. Ethical approval for the study was granted by the Women and Newborn Health Service and the St. John of God Healthcare Human Research Ethics Committees (references RGS0000000309 and #1216, respectively). There were no changes to the methods after trial commencement. Patients were eligible to participate if they were aged ≥ 18 to ≤ 65 years (in accordance with the Nestle Optifast VLCD Clinical Treatment Protocol [15]) with severe obesity (BMI≥ 35 kg/m^2^) and had histologically confirmed Grade 1 endometrioid adenocarcinoma of the uterine corpus on endometrial biopsy reported by a gynaecologic pathologist, clinical stage 1 disease according to the FIGO 2009 staging system, negative metastatic work‐up on CT chest/abdomen/pelvis, and serum CA125 < 35kU/L. Patients were excluded if they had histology showing atypical endometrial hyperplasia, contraindications to VLCD (see Table S1), BMI ≥ 70 kg/m^2^, Eastern Cooperative Oncology Group (ECOG) performance status > 3, were non‐English speaking, already on hormone treatment, wanting fertility preservation, had a synchronous primary cancer, had a significant mental health condition, declined surgery or had significant surgical complexity, or were participating in a competing clinical trial. The study was conducted in accordance with the provisions of the Declaration of Helsinki [21] and Good Clinical Practice guidelines [22] at two tertiary academic gynaecologic cancer centres in Perth, Western Australia. Participants were identified from new patient referrals by lead investigators at each site. Eligible patients were recruited at outpatient clinics or in private consulting rooms. They were provided with information brochures and counselled about the trial by study nurses and site investigators. All participants provided written, informed consent.

At enrolment, participants had a nutritional assessment with a dietitian, baseline fasting blood tests, and anthropometric and blood pressure measurements. All patients were screened with a validated Malnutrition screening tool (MST) by the study dietitian.

Patients had minimum fortnightly contact with the dietitian, plus an additional contact within the first week of starting the study, either face‐to‐face or by telephone. Anthropometric measurements, blood pressure, urinary ketones, and acceptability and tolerability of the diet assessed by the Very Low‐Calorie Questionnaire (VLCD) were recorded fortnightly. The VLCD questionnaire is shown in the Data S2 10.2. These were requested with GPs or GP Nurses for those patients living further from the hospital. Dietetic reviews included providing a supply of Optifast products, 24 h diet recall, review of food diaries, counselling for overcoming barriers and challenges, encouragement for adherence to the Optifast diet, recipes and meal planning ideas, and encouragement for increasing physical activity (although this was not a requirement of the study). The routine use of Preload as part of enhanced recovery after surgery (ERAS) was excluded to prevent fluid changes associated with the cessation of ketosis. On the day of surgery, additional fasting blood tests and measurements were taken. The Charlson comorbidity index [23] was calculated, and data on previous weight loss attempts and pharmacotherapy, surgical and oncologic outcomes were recorded.

### 2.1 Intervention

A minimum of 4 weeks up to a maximum of 6 weeks of VLCD with Optifast was prescribed after discussion between the treating Gynaecologic Oncologist and the patient. Patients were counselled that by enrolling in the trial they may delay the time to surgery and breach the 30‐day boundary in Australia for elective cancer surgery. All patients were required to do the ‘Intensive Phase’ which was 800 kcal (< 3300 kJ) per day containing all recommended daily intake of protein, carbohydrate, essential fatty acids, fibre, vitamins, minerals and trace elements [15]. Total meal replacement with three Optifast VLCD products replaced regular main meals (in the form of shakes, soups, desserts or bars). Protein requirements were calculated using the Optifast protocol and prescribed at 0.8 g/kg/adjusted ideal body weight [15]. Patients with protein requirements > 80 g/day were encouraged to use a fourth Optifast product per day, increasing their daily energy intake to approximately 1000 kcal.

As per the Nestle Optifast VLCD Clinical Treatment Protocol, a minimum of 2 cups of low starch vegetables was allowed per day in addition to 1 teaspoon of oil per day to help the gallbladder contract in the absence of additional fat in the diet [15]. A minimum of 2 L of water and other calorie‐free beverages each day was allowed [15]. Alcohol consumption was discouraged. Normal diet was resumed post‐op with no planned refeeding period. Surgery was performed immediately on cessation of the VLCD and was not contingent on weight loss.

### 2.2 Anthropometric Measurements

Weight was recorded in the clinic by electronic scales with participants wearing light clothing and no shoes. Height was determined with a wall‐mounted stadiometer. Waist circumference was taken at the narrowest point between the lower rib margin and iliac crest, and hip circumference at the widest part over the greater trochanters. Blood pressure was recorded with patients seated.

### 2.3 Blood Tests

Baseline and pre‐operative fasting bloods were performed to assess full blood count, serum electrolytes, uric acid, liver enzymes, albumin, lipid profile, blood glucose, insulin and HbA1c.

### 2.4 Dietary Compliance and Acceptability

Dietary compliance was assessed by fortnightly urinary ketone reagent strips. After the first week of a VLCD, an increase in urinary excretion of ketoacids occurs after increased fat catabolism; therefore, the presence of at least trace urinary ketones was considered to indicate lipolysis and dietary adherence. The very low‐calorie questionnaire was used to record product side effects (nausea/vomiting, bowel changes) and acceptability (taste, hunger, emotional and social eating). Participants were asked to rank these six factors on a 5‐point Likert scale.

### 2.5 Outcomes

The primary outcomes of this study were the safety and feasibility of the intervention. Safety was defined as serious adverse events due to the study intervention that led to study discontinuation or delay in surgery. Feasibility was defined as the ability to recruit 20 participants during the study time frame, with all completing the planned intervention. Secondary outcomes included body weight, anthropometric measures (hip and waist circumference, waist‐hip ratio), blood pressure, biochemistry, estimated blood loss, perioperative complications, length of hospital stay, final tumor stage, grade and histotype.

### 2.6 Sample Size

The sample size for this study was pragmatic. Of approximately 125 patients with endometrial cancer referred to our two centres per year, we conservatively estimated that we would recruit 20 participants over 2 years.

### 2.7 Changes to Protocol/Protocol Violations

Due to the Covid 19 pandemic, the study was put on hold for a 3‐month period in early 2020. Three participants in the study at the time of the pandemic had a shortened duration of Optifast (3 weeks) to facilitate earlier surgical dates before impending theatre closures.

Protocol violations included enrolment of a 67‐year‐old patient, and three participants had mild elevations in serum CA125 at enrolment (values 52, 37, 41kU/L). One patient was enrolled who had previously been a participant in another clinical trial and had failed treatment with a levonorgestrel intrauterine device that was still in situ.

### 2.8 Statistical Analysis

Data were analyzed using IBM SPSS Statistics for Windows, version 27 (IBM Corp., Armonk, N.Y., USA), with an alpha of 0.05 considered statistically significant. Categorical variables were described using frequency and percent, with missing data noted. Continuous scale variables were described using mean and standard deviation and were assessed for normality by the Shapiro Wilk test. Non‐parametric variables were described using median and interquartile range (IQR). Mean pre‐ and post‐intervention weight, body mass index, hip and waist circumference, waist‐hip ratio, and systolic and diastolic blood pressure, fasting glucose, HDL, total cholesterol/HDL ratio, ALT and albumin were compared by the paired‐samples *t*‐test for normally distributed data. Pre‐ and post‐intervention mean fasting insulin, HbA1c, total and LDL cholesterol, triglycerides, AST, GGT and ALP were assessed by the Wilcoxon signed rank test for non‐normally distributed data. Mean scores for each question in the VLCD Questionnaire were compared between week 2 and week 4 and between week 2 and week 6 by the Wilcoxon signed rank test for related samples. Participants' summed scores for hunger were compared with total percentage weight loss by bivariate correlation analysis.

### 2.9 Funding

This study was funded by a research grant from the Australian Society of Gynaecologic Oncologists (ASGO). Optifast was provided at cost–price by Nestle, the manufacturer. Nestle and the funders of the study had no role in study design, data collection, data analysis, data interpretation or writing of the manuscript. The first and senior authors (CA, PC) had full access to the data, vouch for the integrity of the data, and the adherence to the study protocol. All authors are responsible for the final decision to submit for publication. The data were collected by the Principal Investigator (CA) and the dietician at the time points specified in the protocol.

## 3 Results

**FIGURE 1:**
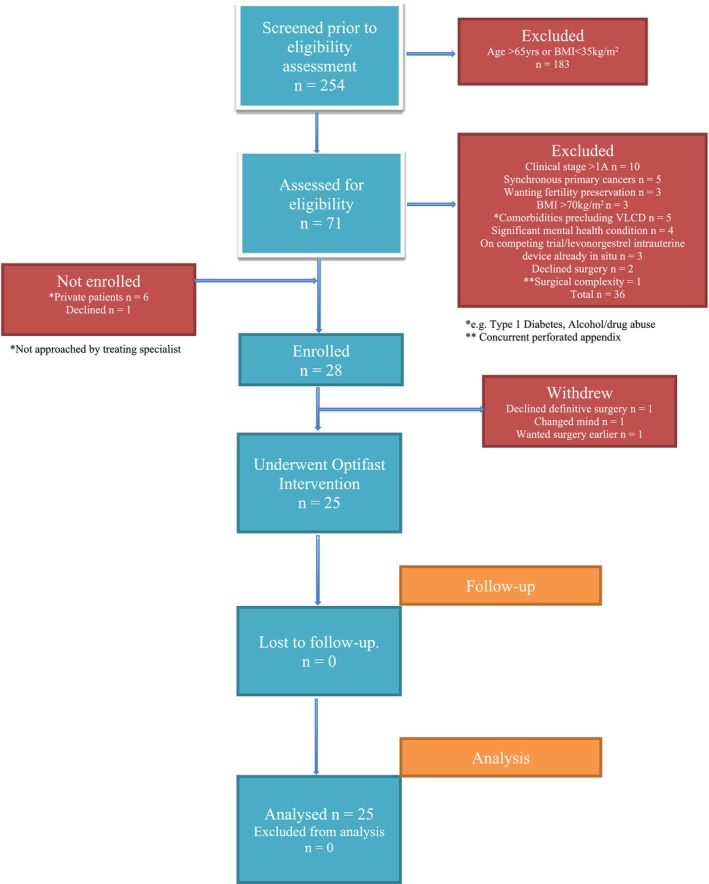
CONSORT flow diagram of optifast study.

Between July 2018 and September 2020, 254 patients with Grade 1 endometrioid histology were screened (Figure 1). Of 71 potentially eligible patients, 28 women were enrolled. Three women withdrew early because of a change of mind, not wanting surgery, or wanting surgery sooner. Twenty‐five women completed the study and were included in the analysis. No participants were lost to follow‐up. Three participants recruited during the Covid pandemic completed 3 weeks. Ten participants completed 4 weeks of the VLCD. Fourteen participants completed 6 weeks. One participant completed 10 weeks of the VLCD.

Table 1 shows the baseline characteristics of the Optifast patient cohort. The mean age of participants at recruitment was 56.4 years (range 43–67 years). Mean weight was 121 kg (range 85–156 kg) and mean BMI was 45.2 (range 35–58). Mean waist and hip circumference were 123 and 141 cm, respectively. Mean Waist‐hip ratio was 0.88. Mean systolic blood pressure (SBP) was 140.1 mmHg and mean diastolic blood pressure (DBP) 83.3 mmHg. Two‐thirds (68%–17/25) of participants were post‐menopausal. Ten of 25 (40%) patients were nulliparous. Charlson comorbidity index was 0 (12%–3/25), 1 (12%–3/25), 2(48%–12/25), 3 (8%–2/25), 4 (20%–5/25) with the main comorbidities being hypertension, type II diabetes, and obstructive sleep apnoea. Sixty percent of patients had previous unsuccessful weight loss attempts with various diets. None had tried pharmacotherapy. On malnutrition screening, all participants scored 0, indicating a low risk of malnutrition. All participants had a baseline albumin recorded.

**TABLE 1:** Baseline participant characteristics.

Variable	*n* (%)	Mean	SD
Age at diagnosis (years)	25 (100)	56.4	6.3
Weight (kg)	25 (100)	121.0	20.4
Height (m)	25 (100)	1.64	0.07
BMI (kg/m^2^)	25 (100)	45.2	7.1
Waist circumference (cm)	25 (100)	123.0	12.7
Hip circumference (cm)	25 (100)	141.0	15.7
Waist/hip ratio	24 (100)	0.88	0.09
Systolic blood pressure (mmHg)	22 (100)	140.1	13.8
Diastolic blood pressure (mmHg)	22 (100)	83.3	10.6
Charlson comorbidity index			
0	3 (12)		
1	3 (12)		
2	12 (48)		
3	2 (8)		
4	5 (20)		
Menopausal status			
Premenopausal	8 (32)		
Postmenopausal	17 (68)		
Area of residence			
Metropolitan	15 (60)		
Rural/remote	10 (40)		
Previous weight loss attempt			
Yes	15 (60)		
No	4 (16)		
Unknown	6 (24)		

Table 2 shows the clinical characteristics of participants pre‐ and post‐intervention. There were significant reductions in weight (mean decrease 8.2 kg SD3.6), BMI (mean decrease 3.1 kg/m^2^ SD1.3), waist circumference (mean decrease 5.7 cm SD6.5), hip circumference (mean decrease 4.5 cm SD4.1) and diastolic blood pressure (mean decrease 10 mmHg SD14.1) (*p* < 0.001 for all). Weight loss ranged from 1.8 (patient on 10 weeks VLCD) to 16.95 kg (patient on 6 weeks VLCD). No significant differences in waist‐to‐hip ratio or systolic blood pressure were observed pre‐ and post‐intervention.

**TABLE 2:** Clinical characteristics of participants pre‐ and post‐very low‐calorie diet.

Variable	Pre‐mean	SD	Post‐mean	SD	Mean difference	SD	95% CI	*p*
Weight (kg)	121.1	20.4	112.9	19.1	−8.2	3.6	6.8–9.7	< 0.001
BMI (kg/m^2^)	45.2	7.1	42.1	6.8	−3.1	1.3	2.5–3.6	< 0.001
Waist circumference (cm)	123.0	12.7	117.3	13.6	−5.7	6.5	3.0–8.5	< 0.001
Hip circumference (cm)	141.0	15.7	136.5	13.8	−4.5	4.1	2.7–6.2	< 0.001
Waist/hip ratio	0.88	0.09	0.86	0.08	−0.02	0.05	−0.0–0.0	0.12
Systolic blood pressure (mmHg)	140.1	13.8	133.4	14.9	−6.7	19.6	−2.0 –15.4	0.13
Diastolic blood pressure (mmHg)	83.3	10.6	73.2	10.0	−10.0	14.1	3.8–16.3	0.003

*Note:* There were no missing data.

Abbreviations: BMI, body mass index (kg/m^2^); mmHg, millimetres mercury.

The biochemical profiles of the patients pre and post VLCD are shown in Table 3. Significant decreases in HbA1C, HDL and total cholesterol and ALP were observed following the intervention, but no decreases in fasting glucose, insulin or LDL cholesterol (Table 3). Serum albumin increased on the VLCD (*p* < 0.001) as did AST (*p* < 0.01) (Table 3). There were no significant abnormalities in electrolytes, full blood count or uric acid.

**TABLE 3:** Biochemical profiles of participants pre‐ and post‐very low‐calorie diet.

Variable	Pre‐mean (SD)	Pre‐median (IQR)	Post‐mean (SD)	Post‐median (IQR)	Mean difference	Median difference	*p*
Fasting glucose (mmol/L)	5.76 (1.0)		5.73 (1.0)		−0.03		0.83
Fasting insulin (μU/mL)		12.5 (14)		11.5 (8)		−1.0	0.14^b^
HBA1C (%)		5.8 (0.5)		5.6 (0.6)		−0.2	0.01^b^
Total cholesterol (mmol/L)		4.85 (1.6)		4.5 (1.3)		−0.35	< 0.01^b^
HDL (mmol/L)	1.2 (0.3)		1.1 (0.1)		−0.1		0.02
LDL (mmol/L)		2.8 (1.3)		2.8 (0.9)	0.0		0.14^a^ ^,^ ^b^
Total cholesterol/HDL ratio		4.1 (1.7)		3.95 (1.6)	−0.15		< 0.56^a^ ^,^ ^b^
Triglycerides (mmol/l)		1.2 (0.7)		1.1 (0.7)	−0.1		0.20^b^
ALT (U/L)	30.5 (12.1)		33.9 (13)		+3.4		0.10
AST (U/L)		24.5 (8)		32.5 (13)		+8	< 0.01^a^,^b^
GGT (U/L)		34 (25)		33 (17)		−1	0.19^a^,^b^
ALP (U/L)		84 (18)		75 (20)		−9	< 0.01^a^,^b^
Albumin (g/L)	41.8		44.3		+2.5		< 0.001

*Note:* There were no missing data.

Abbreviations: ALP, alkaline phosphatase; ALT, alanine aminotransferase; AST, aspartate transferase; GGT, gamma‐glutamyl transferase; HBA1C, glycosylated haemoglobin; HDL, high density lipoprotein; LDL low density lipoprotein.

^a^ Not normally distributed as per Shapiro–Wilk test.

^b^ Wilcoxon signed‐rank test.

All patients underwent laparoscopic surgery. Three patients had a mini laparotomy to retrieve the uterus to avoid morcellation. Twenty patients had a total hysterectomy and bilateral salpingo‐oophorectomy, and bilateral sentinel lymph node biopsies and five patients had a total hysterectomy and bilateral salpingo‐oophorectomy. Clavien Dindo grade 1 complications occurred in 32% of participants and grade 2 complications occurred in 8% (2/25) (Table 4). There were no delays as patients were given a surgical date at enrollment.

**TABLE 4:** Clinical and histopathological variables.

Variable	*n* (%)
Estimated blood loss (ml)	25 (100)
< 50 mL	4 (16)
50–100 mL	4 (16)
> 100 mL	13 (54)
Missing	4 (16)
Surgical complications	25 (100)
None	15 (60)
Grade 1	8 (32)
Grade 2	2 (8)
Length of hospital stay (nights)	25 (100)
1	1 (4)
2	13 (52)
3	5 (20)
4	2 (8)
Missing	4 (16)
Final FIGO 2009 stage and grade	
IA, grade 1	22 (88)
IA, mixed grade (80% grade1, 20% grade 3 clear cell)	1 (4)
IB, grade 1	1 (4)
II, grade 1	1 (4)

*Note:* Surgical complications (Clavien–Dindo Classification). Grade 1—any deviation from normal postoperative course without the need for pharmacological treatment or surgical, endoscopic and radiologic interventions. Grade 2—requiring pharmacological treatment with drugs other than those allowed for grade 1 complications. Blood transfusions and total parenteral nutrition are also included.

Abbreviation: FIGO, International Federation of Gynecology and Obstetrics.

Eighty‐eight percent (22/25) of participants had FIGO 2009 Stage 1A Grade 1 endometrioid endometrial adenocarcinoma. One patient had a stage 1B disease, another had microscopic stage II disease, and in one patient, the histopathology was upgraded to a mixed grade 1 (80%) and grade 3 clear cell (20%) carcinoma (Table 4).

### 3.1 Patient Acceptability and Compliance

Dietary compliance was assessed using fortnightly urinary ketones. The presence of at least trace ketones was considered to indicate dietary adherence. Compliance at 2, 4 and 6 weeks was 61.9%, 40% and 50%, respectively.

Significant differences were observed for Question 2 ‘how hungry are you?’ between week 2 and week 4 (*p* = 0.02) and between week 2 and week 6 (*p* = 0.05) (Table 5). No significant differences were found for the other questions between week 2 and weeks 4 and 6. No correlation was found between participants' summed scores for hunger and total percentage weight loss (Pearson correlation = 0.04; *p* = 0.85). The product taste was considered acceptable to highly acceptable and compliance was high with minimal emotional and social eating reported.

**TABLE 5:** Very low‐calorie questionnaire responses.

	Week 2	Week 4	Week 6	*p* values	*p* values
Question	*n* = 25 (100%)	*n* = 25 (100%)	*n* = 14 (100%)	Week 2 vs week 4	Week 2 vs week 6
How do you rate the product taste?					
Tolerable	1 (4)	1 (4)	1 (7)	0.18	0.22
Acceptable	9 (36)	10 (40)	4 (29)		
Highly acceptable	15 (60)	14 (56)	9 (64)		
How hungry are you?					
Most days	0 (0)	1 (4)	1 (7)	0.02	0.05
Some of the time	10 (40)	2 (8)	2 (14)		
Occasional	9 (36)	12 (48)	5 (36)		
No hunger	6 (24)	10 (40)	6 (43)		
How much nausea/vomiting do you have?					
2‐3 times per week	1 (4)	0 (0)	0 (0)	0.5	0.75
≤ 1 x per week	1 (4)	0 (0)	1 (7)		
None	23 (92)	25 (100)	13 (93)		
How well are your bowels working compared to what is normal for you?					
No bowel motion for past 4 days	0 (0)	0 (0)	0 (0)	1	0.13
No bowel motion for past 2‐3 days	3 (12)	3 (12)	4 (29)		
Normal	18 (72)	18 (72)	10 (71)		
Increased frequency	1 (4)	2 (8)	0 (0)		
Diarrhoea	3 (12)	2 (8)	0 (0)		
How often are you emotional eating?					
Daily	0	0	1 (7)	0.83	1
2‐3 x per week	3 (12)	3 (12)	0 (0)		
≤ 1 x per week	5 (20)	7 (28)	5 (36)		
None	17 (68)	15 (60)	8 (57)		
How often are you social eating?					
Daily	0 (0)	0 (0)	0 (0)	0.13	0.63
2‐3 x per week	1 (4)	4 (16)	2 (14)		
≤ 1 x per week	8 (32)	7 (28)	5 (36)		
None	16 (64)	14 (56)	7 (50)		

*Note:* total number of participants at 6 weeks was 14 due to completion of the intervention prior to 6 weeks in 11 patients.

One patient had a flare of gout in the first 2 weeks of VLCD and was managed with colchicine and allopurinol. This was associated with a weight loss of 6.4 kg in 2 weeks. There were no withdrawals due to adverse events.

## 4 Discussion

A 4–6‐week pre‐operative VLCD in severely obese endometrial cancer patients appeared to be feasible, acceptable, and safe. There were significant decreases in weight, BMI, waist and hip circumference, and diastolic blood pressure, and all participants underwent definitive surgery without serious adverse events. VLCD appears to be oncologically safe with 88% of patients having 2009 FIGO stage 1A Grade 1 endometrioid adenocarcinoma on their final histology, representing the earliest stage and lowest risk histology endometrial cancer.

Our findings are consistent with those of studies in the bariatric and general surgical literature [1, 2, 3, 4, 5, 6]. An energy‐restricted diet is often prescribed prior to bariatric surgery to reduce weight and liver volume [16]. A systematic review showed that a low‐calorie diet (LCD) was effective in liver volume reduction (12%–27%) and weight loss (4%–17%), particularly during the first weeks with acceptable compliance, which is consistent with the weight loss observed in our study [5]. Whilst we appreciate that a reduction in liver volume does not impact the technical complexity of laparoscopic pelvic surgery, there would be an anaesthetic benefit to less mechanical compression of the lungs when patients are in a steep Trendelenburg position with a pneumoperitoneum [24]. A retrospective mixed methods study of 78 patients undergoing a range of elective surgical procedures that included 47 gynaecologic operations reported that a dietitian‐led VLCD achieved sufficient weight loss to facilitate elective surgery for most patients, and surgeons found that VLCD‐based treatment made surgery easier [4]. A systematic review that included nine studies and a total of 849 patients prior to bariatric surgery found VLCD treatment led to significant weight loss and liver volume reduction, although the impact of VLCD on perioperative risks was unclear [5]. To our knowledge, no studies have evaluated VLCDs in severely obese patients with endometrial cancer prior to surgery.

The mean waist circumference of our cohort was 123 cm. A waist circumference > 80 cm in women increases the risk of metabolic complications, and > 88 cm substantially increases this risk [16]. Whilst there was no significant change in mean waist– hip ratio between baseline and completion, study participants had a mean waist–hip ratio of 0.88. A ratio of > 0.85 is abnormal and shows that the participants are at substantial risk of metabolic complications, cardiovascular disease and all‐cause mortality [25]. The use of specialist body fat percentage scales, which look at overall body fat, was decided against in this study. Instead, waist and hip circumference were used to look at where patients store their ‘dangerous’ fat and therefore increase their metabolic disease risk.

A significant decrease in HbA1c was observed, consistent with the findings of the study by Colles et al. that reported significant reductions in fasting insulin and HbA1c after a 12‐week VLCD [16]. AST levels significantly increased after the dietary intervention. In obese women, mild transient increases in AST are observed immediately after VLCD and are multifactorial in origin but considered likely benign as long as they remain transient [26]. HDL levels were also noted to decrease, which is normal with active weight loss using a VLCD, but these are known to return to pre‐VLCD levels or improve overall with the weight maintenance phase [27].

All surgery was performed laparoscopically. It should be noted that there is no public access to robotic surgery for obese endometrial cancer patients in Western Australia. There were no conversions to laparotomy and no major complications. Fifty‐six percent (14/25) of patients stayed 1–2 nights. Twenty‐eight percent (7/25) of patients had a hospital stay of 3 days or more; however, 40% (10/25) of the study cohort lived in rural/remote regions, so prolonged admissions were likely due to the unique demographics of Western Australia and social factors. Our findings suggest that blood loss volume was small given that no patient required a blood transfusion or iron infusion.

Previous literature suggests a survival disadvantage to delaying time from diagnosis to surgery by 6 weeks, and this is an important consideration when deciding on the optimal duration of a dietary or weight loss intervention in cancer patients [28, 29].

In this study, a 4–6‐week time frame was proposed to achieve maximum reductions in body weight without compromising compliance and acceptability, as a previous study found that hunger and emotional eating increased, and taste acceptability decreased between week 4 and week 10, likely due to boredom with ongoing dietary restriction [16]. The product taste was considered acceptable to highly acceptable, and compliance was high, with minimal emotional and social eating reported. Urine ketone measurements as a surrogate for compliance would suggest less dietary compliance, with only 61.9% of participants having at least trace urinary ketones at week 2 of the VLCD, 40% at week 4 and 50% at week 6. It was notable that all patients lost weight regardless of the absence of urinary ketones.

Strengths of the study are its prospective design and pragmatic inclusion criteria. To our knowledge, this is the first study to evaluate a VLCD in the setting of severely obese endometrial cancer patients prior to surgery. Limitations that should be acknowledged include the single‐arm observational design that aimed to assess feasibility and was not adequately powered to evaluate perioperative complication rates. A further limitation was the dietetic support that was integral to the successful delivery of the intervention and hence, the findings may not be applicable in other settings. Patients with atypical endometrial hyperplasia were excluded from the study as we wanted to include a homogenous ‘cancer only’ population. This decision was also pragmatic because, in Western Australia, not all patients with atypical endometrial hyperplasia are operated on by Certified Gynaecologic Oncologists. Patients over the age 65 years were also excluded as per the Nestle Optifast VLCD Clinical Treatment Protocol, as the metabolic and physical adaptations to intensive VLCDs are reduced in this age group. This may limit the external validity of our findings.

An additional limitation of the study was that the time from diagnosis to surgery was not recorded. However, our center follows the Optimal care pathway for women with endometrial cancer who are seen within 4 weeks of receipt of referral and undergo surgery within 4 weeks of the multidisciplinary tumour board meeting [30]. In Western Australia, there is no unified patient electronic medical record to enable access to data regarding emergency department presentations, unscheduled general/family practitioner visits, or rural/regional hospital admissions following discharge from the tertiary metropolitan hospital. Forty percent of our study participants lived in rural or remote locations, and we did not have access to their local hospital or general/family practitioner data, which is a further limitation of the study. Due to its single‐arm observational design, we were unable to conclude from our study whether the VLCD intervention reduced surgical risk, and this should be the subject of future randomised trials. It is also possible that because Optifast was provided to study participants at no cost that our findings may not be applicable in settings where patients might be required to pay for a VLCD.

The hypothesis that preoperative VLCD in obese endometrial cancer patients improves surgical outcomes should be tested in large multicentre prospective studies. During the conduct of the current study, more recent interventions such as glucagon‐like peptide‐1 (GLP‐1) agonists were not readily available in Australia, although they are now being used more widely, and their role in the low‐risk endometrial cancer population requires evaluation [31].

## 5 Conclusion

In this study, a pre‐operative VLCD in severely obese patients with low‐grade, clinically early‐stage endometrial cancer was feasible, well tolerated, and safe. Significant reductions in weight, BMI, waist and hip circumference, diastolic blood pressure, HbA1C, and total cholesterol were observed. Patients on a 4–6 week VLCD did not appear to be at an oncologic disadvantage, with 88% (22/25) patients having FIGO 2009 Stage 1A Grade 1 endometrioid endometrial adenocarcinoma on final staging, which carries a very low risk of recurrence and has an excellent prognosis.

## Author Contributions

**Chloe Ayres:** conceptualization, methodology, formal analysis, investigation, funding acquisition, writing – original draft. **Hanna Burbidge:** conceptualization, methodology, writing – review and editing. **Jayna Garratt:** methodology, investigation, writing – review and editing. **Ganendra Raj Mohan:** conceptualization, methodology, writing – review and editing. **Yee Leung:** methodology, investigation, writing – review and editing. **Stephanie Jeffares:** methodology, project administration, writing – review and editing. **Sanela Bilic:** methodology, project administration, writing – review and editing. **Paul A. Cohen:** conceptualization, methodology, formal analysis, investigation, writing – original draft.

## Disclosure

PAC declares speakers' honoraria from Astra Zeneca and MSD, Advisory board Astra Zeneca, and stock and consultancy fees from Reliis Ltd.

## Ethics Statement

Ethical approval for the study was granted by the Women and Newborn Health Service and the St. John of God Healthcare Human Research Ethics Committees (references RGS0000000309 and #1216 respectively).

## Consent

All participants provided written, informed consent.

## Conflicts of Interest

The authors declare no conflicts of interest.

## Supporting information


**Table S1:** Supporting Information.

**Data S2:** Supporting Information.

## Data Availability

The data that support the findings of this study are available from the corresponding author upon reasonable request.
