# Supplementary material for: A Single Arm Pilot Observational Study to Evaluate the Safety and Feasibility of a Pre‐Operative Very Low Calorie Diet in Severely Obese Patients With Endometrial Cancer
Source: Cancer Rep (Hoboken). 2025 Apr 3;8(4):e70172. doi: 10.1002/cnr2.70172 (PMC11965880; doi:10.1002/cnr2.70172)
Supplement: Supplementary file 1 — Table S1: Supporting Information. [file CNR2-8-e70172-s001.docx]

**Supplementary Table**

| **Contraindications to VLCD** | **Precautions to VLCD** |
| --- | --- |
| Normal weight (BMI <25m^2^) | Age >65 years |
| Pregnancy | History of severe psychological disturbance, alcoholism or drug abuse |
| Lactation | Cholelithiasis, Pancreatitis, Gout |
| Children <18 years | Liver enzymes abnormalities |
| Presence of porphyria | Electrolyte disturbance (especially if on diuretics) |
| Recent myocardial infarct or unstable angina | Medications  -Oral hypoglycaemics (sulfonylureas)/short and long acting insulin for type 1 or 2 diabetes  -Warfarin  -Diuretics  -Lithium  -Anticonvulsants  -Corticosteroids |
| Severe or advanced renal or liver disease |  |

*Modified from the Optifast® VLCD™ Clinical Treatment Protocol available at: https://www.nestlehealthconnect.com.au/education-centre/obesity/optifast-vlcd-clinical-treatment-protocol
