# Supplementary material for: A Single Arm Pilot Observational Study to Evaluate the Safety and Feasibility of a Pre‐Operative Very Low Calorie Diet in Severely Obese Patients With Endometrial Cancer
Source: Cancer Rep (Hoboken). 2025 Apr 3;8(4):e70172. doi: 10.1002/cnr2.70172 (PMC11965880; doi:10.1002/cnr2.70172)
Supplement: Supplementary file 2 — Data S2: Supporting Information. [file CNR2-8-e70172-s002.docx]

**The Case for Pre-operative Very Low Calorie Diets (VLCDs) in Obese Endometrial Cancer Patients.**

Version 3

13.08.2017

SYNOPSIS

Protocol title: The Case for Pre-operative Very Low Calorie Diets (VLCDs) in Obese Endometrial Cancer Patients.

Short title: Pre-operative Optifast in Obese Endometrial Cancer Patients.

Protocol version: 3

LIST OF INVESTIGATORS

Principal Investigator: Dr Chloe Ayres

Organisation: King Edward Memorial Hospital

Address: 374 Bagot Road, Subiaco

Telephone: 08 6458 2222

Email: chloe.ayres@health.wa.gov.au

Principal Investigator: Dr Paul Cohen

Organisation: St John Of God Subiaco Hospital

Address: 12 Salvado Road, Subiaco, WA 6008

Telephone: 08 9382 6111

Email: paul.cohen@uwa.edu.au

Associate Investigator: Dr Ganendra Raj Mohan

Organisation: St John Of God Subiaco Hospital

Address: 12 Salvado Road, Subiaco, WA 6008

Telephone: 08 9382 6111

Email: raj.ganendra@gmail.com

Associate Investigator: Professor Yee Leung

Organisation: King Edward Memorial Hospital

Address: 374 Bagot Road, Subiaco

Telephone: 08 6458 2222

Email: yee.leung@health.wa.gov.au

SUMMARY

Study title: The Case for Pre-operative Very Low Calorie Diets (VLCDs) in Obese Endometrial Cancer Patients.

Short title: Pre-operative Optifast in Obese Endometrial Cancer Patients.

Protocol version: 1

Objectives

Primary objective:

-To evaluate the feasibility of a 4-6 week pre-operative very low calorie diet (VLCD) with Optifast® in severely obese (BMI ≥ 35) low risk endometrial cancer patients (Grade 1 endometrioid adenocarcinoma with no or minimal myometrial invasion) planned to undergo a laparoscopic hysterectomy. Dietary compliance will be assessed by urinary ketone reagent strips.

Secondary objectives:

-To monitor changes over the 4-6 week VLCD period in body weight, BMI, anthropometric measures (hip and waist circumference), clinical variables of blood pressure and biochemical variables (fasting blood sugar and cholesterol).

-To evaluate tolerability of the VLCD using a validated qualitative questionnaire.

-To collect data on surgical outcomes.

Study design: This is a single institution (St John of God Subiaco Hospital) prospective observational study of women in Western Australia diagnosed with low risk endometrial cancer, BMI ≥35, planned to undergo a laparoscopic hysterectomy.

Planned sample size and selection criteria: The study will aim to recruit 20 patients over a 12 month period and will include women recommended to have Optifast as part of their pre-operative preparation for surgery.

Study procedure: Women aged 18-65 years with a low risk endometrial cancer of the uterus with a BMI ≥35 who have been recommended to have a very low calorie diet prior to planned surgery will be invited by their Gynaecological Oncologist to participate in this observational study. They will be given the relevant information package and consent forms to read at their initial consultation. If they agree to consent to the study, they will have an initial nutrition assessment with a dietitian. This will include performing baseline fasting blood tests, anthropometric and blood pressure measurements, in addition to the provision of written and verbal information regarding following a VLCD pre-operatively. Patients will then be required to have fortnightly contact with the dietitian, either face-to-face or over the phone, for 4-6 weeks to facilitate dietary adherence and success. Urinary ketones will be measured fortnightly to assess dietary compliance. The 4-6 week time frame will be at the discretion of the treating Gynaecological Oncologist. Clinical data will be recorded and the data collected will be de-identified. On the day of surgery additional fasting blood tests will be collected in the operating theatre. Australian Clinical Labs will be responsible for processing all blood samples. Biospecimens obtained at surgery will undergo routine cytology and histopathology by the Anatomical Pathology Department at Australian Clinical Labs. Operative data will be recorded. Tumour stage, grade and ongoing management plan will be ascertained from the minutes of the weekly combined King Edward Memorial Hospital and St John of God Subiaco Hospital tumour conference. Additional information on post-operative course will be obtained from the patient’s discharge summary.

Statistical considerations: The sample size for this observational, proof of concept study is pragmatic and analysis of will be descriptive. The findings of this study will facilitate calculation of the sample size calculation for a subsequent, appropriately powered, randomized controlled trial. Data analysis will be performed using the statistical software IBM SPSS Statistics Version 20.

Duration of the Study: Time taken to enroll patients and collect clinical data is estimated to take 12-18 months. Complete analysis of all data may take up to 2 years.

**TABLE OF CONTENTS**

1. BACKGROUND 6
   1. Study Background
   2. Rationale for performing the study
2. STUDY OBJECTIVES 8
   1. Primary Objective
   2. Secondary Objectives
3. STUDY DESIGN 8
   1. Design and Study groups
   2. Number of Participants
   3. Number of Centres
   4. Duration
4. PARTICIPANT SELECTION 11
   1. Inclusion Criteria
   2. Exclusion Criteria
5. STUDY OUTLINE 12
   1. Study Flow Chart
   2. Investigation Plan
   3. Study Procedure Risks
   4. Recruitment and Screening
   5. Informed Consent Process
   6. Enrolment Procedure
6. SAFETY 15
   1. Study Processes
   2. Data Safety and Monitoring Board
   3. References in National and International guidelines on research in humans
   4. Early study termination
7. STATISTICAL CONSIDERATIONS 15
   1. Sample size calculation and analysis plan
8. STORAGE AND ARCHIVING OF STUDY DOCUMENTS 16
9. REFERENCES 16
10. APPENDICES 19
    1. Initial Visit History Form
    2. Patient VLCD questionnaire

1. BACKGROUND

1.1 Study Background

Endometrial cancer is the most common gynaecological malignancy in Australia with 2652 new cases estimated to be diagnosed this year^1^. Over the past 30 years, the incidence and mortality of endometrial cancer has continued to rise^2,3^ coinciding with a national epidemic of obesity. Endometrial cancer ranks highest amongst all cancers in its association with obesity - every 5mg/m^2^ increase in BMI confers a 1.6 fold increased risk of endometrial cancer^4^. According to the Australian Bureau of Statistics, 56% of Australian adult women are now overweight or obese based on body mass index with 28% classified as obese (BMI≥30)^5.^

Fortunately studies have shown an inverse relationship between BMI and tumor biology. Most obese women with endometrial cancer have a tendency towards a slightly more favorable histopathological phenotype with MSI (microsatellite instability) negative, early stage and lower grade tumours^6-8.^

Surgery is the mainstay of endometrial cancer treatment, which includes the removal of the uterus, fallopian tubes, ovaries, with pelvic and para-aortic lymphadenectomy as necessary for surgical staging and management planning. Obesity and its associated fat deposition posess significant challenges to the performance of safe surgery by any technique, whether open or minimally invasive.

Minimally invasive surgery is now recognized as a safe and feasible surgical alternative to laparotomy for morbidly obese women with endometrial cancer ^9,10^. Some studies have shown increased conversion rates^11,12^ and decreased success in obtaining pelvic and para-aortic lymph nodes in obese women^12-14^ however other smaller retrospective studies have shown comparable conversion rates to women of lower BMI^15-17^ and successful pelvic lymphadenectomy^18^. Some studies even show superiority of a laparoscopic approach in regards to surgical complications in the morbidly obese^19-21^.

Despite improvements in anaesthesiology and surgical techniques obesity remains a risk factor for surgery and in some cases delaying surgery until a patient has lost weight to improve peri-operative outcomes and performance status may be advisable.

Non-surgical treatment of low-grade endometrial malignancies with morbid obesity and comorbidities has been directed at restoring the oestrogen-progesterone balance, typically through the addition of progestins. This strategy is often effective but commonly associated with depression and secondary weight gain, which are problematic in the obese. There are no large published case series however reporting on such medical management as primary treatment (feMMe study in progress) and the literature is often focused on younger women who desire fertility preservation^33^ which makes it difficult to apply to the older obese endometrial cancer population. Furthermore these therapies do little to relieve the cardiovascular and endocrine consequences of morbid obesity^37^.

Very Low Calorie Diets (VLCDs), however, can provide safe rapid weight loss in the period immediately preceding surgery and may reduce the perceived perioperative difficulties associated with anaesthetic and surgical techniques. This method of short-term dietary therapy is often overlooked despite being an evidence-based practice supported by the Dietitians Association of Australia (DAA). VLCDs provide 800kcal per day and rank second after bariatric surgery in their ability to help overweight or obese adults lose weight (14.7% mean weight loss at 4-20 weeks) (NHMRC 2006). Patients with a BMI ≥35 can be expected to lose 1.5-2.5kg per week^23^.

VLCDs safely and effectively achieve significant reductions in body weight, liver volume and visceral/subcutaneous adipose tissue. Interestingly 80% of the reduction in liver volume occurs in the first 2 weeks of a VLCD^22^ (see Figure 1). Many of the metabolic and physiologic effects of VLCDs are also beneficial, such as improvement in insulin sensitivity and fasting plasma glucose levels enabling a reduction or even cessation of diabetic medications, lowering of blood pressure and serum triglyceride values ^23,24^. VLCDs are also conducive to ketosis, which can help suppress hunger and preserve lean muscle tissue^23^.


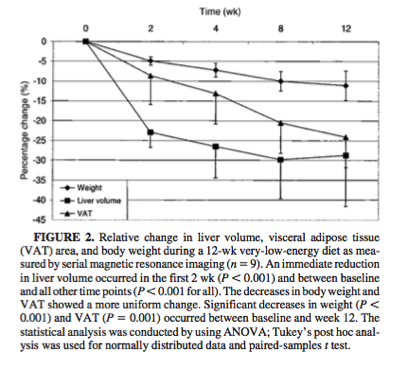


Figure 1: Relative changes in liver volume, visceral adipose tissue and body weight during a 12-week very low calorie diet as measured by serial MRI^22^.

Pre-operative VLCDs such as Optifast^®^ have been used successfully to reduce the perceived technical difficulty and perioperative complications in bariatric surgery, general surgery and more recently in laparoscopic cholecystectomy^25-27^. VLCDs prior to bariatric surgery have well-established benefits. In a multicentre randomized trial, Van Niewenhove et al^26^ investigated the impact of a 2 week preoperative VLCD before laparoscopic gastric bypass surgery in 273 patients with a BMI ≥35. 10.7% of the patients in the VLCD group were not able to complete the diet due to intolerance or non-compliance, however, those who adhered to the VLCD lost 4.9kg compared to 0.4kg in the control group (P <0.001). The surgeons’ perceived difficulty of the procedure (using visual analog score) was also lower in the VLCD group as was the number of post-operative complications; in particular infections at the 30-day follow. Pekkarinen^25^ examined the suitability of a 7-24 week VLCD in general surgical patients with a BMI ≥35 whose elective surgery was postponed because of their obesity. Two patients discontinued and the mean weight loss of the remaining 28 patients was 19.6kg (15% of initial weight). Remarkably in four patients, an operation was no longer necessary after weight loss. More recently, the effectiveness of a 2-week preoperative VLCD in 38 laparoscopic cholecystectomy patients has been investigated^27^. Even though only 32% (6/19) of obese patients (BMI >30) adhered to the VLCD, with a mean weight loss of 2.75kg, the technical difficulty of both the liver retraction and dissection of Calot’s triangle was reduced in those obese patients who adhered to the VLCD compared to the non-compliant obese group. This was thought to be the result of the reduction in liver volume caused by caloric restriction. This was in turn postulated to reduce complications such as bile duct injury.

The compliance rate for short term VLCD’s of 2-24 weeks duration is surprisingly high at 86-96%^22,24-25,31^. Previous studies of VLCDs have shown that hunger and emotional eating increased, and taste acceptability decreased significantly between week 4 and week 10, most likely related to boredom and fatigue with the ongoing dietary restriction^22^. Accordingly, a 6-week time frame has been proposed to achieve maximal liver volume reduction and significant reductions in VAT and body weight without compromising compliance and acceptibility^22^.

VLCDs such as Optifast are routinely employed pre-operatively in many Gynaecological Cancer Units across Australia in the low risk obese endometrial cancer population. However, despite their growing popularity, the feasibility, tolerability and efficacy of these diets has not been investigated in this patient group. A 4-6 week time frame seems reasonable to achieve the greatest benefit safely without delaying surgery or compromising patient adherence and tolerability.

**1.2 Rationale for performing the study**

The rationale of the study is to document the feasibility, tolerability and efficacy of a Very Low Calorie Diet (VLCD) with Optifast in obese endometrial cancer patients by assessing compliance and weight loss. A secondary objective is to measure patients’ metabolic profiles prior to, and following, the intervention to provide data to inform further research.

**2 STUDY OBJECTIVES**

**2.1 Primary Outcome**

The number/percentage of patients with urinary ketone reagent testing suggesting dietary compliance with the 4-6 week very low calorie diet (Optifast) will be the primary outcome measure.

**2.2 Secondary Outcomes**

Secondary outcomes will include:

-Monitoring changes over the 4-6 week VLCD period in body weight, BMI, anthropometric measures (hip and waist circumference), blood pressure and serum biochemistry.

-Evaluating patient tolerability of the VLCD using a validated qualitative questionnaire.

-Collecting data on surgical outcomes.

**3 STUDY DESIGN**

**3.1 Design and study group**

This is a prospective observational study investigating obese (BMI ≥ 35) women with low risk (early stage, low grade endometrioid histology) endometrial cancer planned to undergo laparoscopic hysterectomy.

In addition to the routine information obtained from the initial visit history form (see appendices 10.1), the following information will be recorded:

- Geographical location (rural or local)
- Histology at diagnosis
- Any current non-surgical treatment for endometrial cancer (e.g. Mirena or Provera)
- Any previous history of weight loss, pharmacotherapy or enrolment in weight control programs and amount of weight lost and for how long

The pre-operative Very Low Calorie Diet will be with Optifast® and involves a 4-6 week ‘Intensive Phase’, 800 kcal (<3,300kJ) per day containing all RDI of protein, carbohydrate, essential fatty acids, fibre, vitamins, minerals and trace elements. 3 Optifast VLCD products must replace regular main meals (shakes, soups, desserts or bars). A Minimum of 2 cups of low starch vegetables are allowed per day in addition to 1tsp oil per day to help the gallbladder contract in the absence of additional fat in the diet. A minimum of 2L water and other calorie free beverages each day is allowed. Alcohol consumption is discouraged during the VLCD, however, if this is too difficult for patients, then 1-3 standard drinks per week is the maximum allowed.

As part of this particular research project, participants will have their dietary compliance assessed by urine tests, tolerability of the Optifast diet assessed by questionnaires, additional body measurements and blood tests performed.

Urinary ketone reagent strips will be used to assess dietary compliance. After the first week of a VLCD, an increase in the urinary excretion of ketoacids occurs subsequent to increased fat catabolism; therefore the presence of at least trace ketones in the urine is considered to indicate net lipolysis and dietary adherence^22,30^. Urine samples are to be collected fortnightly at the dietitian or local GP consultation. The absence of ketones is scored 0, trace 1, small amount 2, moderate amount 3. Ketone scores determined at visits every 2 weeks will be summed for each patient.

A validated questionnaire of product side effects and acceptability^22^ will be used. Subjects will be asked to rate 6 factors of product taste, hunger, nausea/vomiting, bowel function, emotional eating and social eating on a 5 point Likert scale (see appendices 10.2).

Anthropometric measurements of height (using a wall-mounted stadiometre), weight (wearing light clothing, no shoes), BMI (weight kg/height m^2^), waist circumference (narrowest point between low rib margin and iliac crest) and hip circumference (widest point over greater trochanters) will be recorded at baseline and fortnightly preferably by the same clinician or their appropriate delegate. Seated blood pressure will be measured at baseline and fortnightly. Blood tests will be taken at baseline and again on the day of surgery (after 4-6 weeks of Optifast) including FBC, EUC, LFTs, CMP, uric acid, CA125, iron studies, fasting lipid profile (Total cholesterol, LDL, HDL, TG), fasting glucose metabolism (plasma glucose, plasma insulin, HBA1C) and 25OHVitD.

All patients will be given instructions on pre operative fasting and be allowed clear fluids up to 2 hours and solids up until 6 hours prior to induction of anaesthetic as per ERAS recommendations. Randomized controlled trials on mechanical bowel preparation have shown no improvement in surgical exposure or bowel handling in laparoscopic gynaecologic procedures^34-35^.

For the laparoscopic hysterectomy, all patients will receive 2g Cephazolin and 500mg metronidazole 15-60 minutes prior to skin incision (as per eTG 2006). Clindamycin (or Lincomycin) 600mg can be used for patients who are allergic to penicillin.

Additional information will be recorded post operatively on:

- Tumour stage, grade, histological type, degree of myoinvasion
- Lymphadenectomy performed – Yes or No
  - Pelvic vs. para-aortic nodes
  - Sentinel lymph node biopsy vs. debulking of enlarged nodes vs. full pelvic lymph node dissection
  - Number of nodes removed
  - Number of nodes positive
- Adjuvant treatment recommendation from multidisciplinary meeting/tumour board
- ASA and Anaesthetic time
- Operating time
- Surgical complications – urinary tract, vascular or bowel injury; cardiac/pulmonary complications; wound breakdown; infection; estimated blood loss
- Length of hospital stay

Post operatively all patients will returns to a normal diet. There will be no planned refeeding period. They will be offered a follow up with dietitian 1 month post operatively and either adopt the Optifast® program with progression to ‘Maintenance Phase’ or a healthy lower calorie diet. Referral to community/private dietitian will be provided beyond 1 year.

**3.2 Number of Participants**

We aim to recruit twenty women.

**3.3 Number of Centres**

St John of God Subiaco Hospital will be the only participating centre.

**3.4 Duration of study**

We plan to commence this study in January 2018, following ethical and governance approval. The anticipated time period for patient recruitment phase and data collection is 24 months. Data analysis would take an extra 6 months. The study will aim to be completed by July 2020.

**4 PARTICIPANT SELECTION**

**Patients receiving Optifast as part of standard of care must meet the following criteria.**

**4.1 Inclusion Criteria**

- Recommended by treating Gynaecological Oncologist to have VLCD prior to surgery
- Age ≥ 18 and ≤65 (outside these ranges, metabolic and physiologic adaptations to intensive diets are decreased)
- BMI ≥ 35
- Histologically confirmed grade 1 endometrioid adenocarcinoma on uterine curetting or endometrial biopsy (pipelle)
- No extra-uterine disease on pre-operative imaging of chest, abdomen or pelvis (any imaging modality allowed - X-ray, CT, MRI, PET)
- Patient wishes to lose weight
- Suitable for laparoscopic hysterectomy at surgeon’s discretion
- Ability to understand study requirements and attend regular appointments with dietitian or GP locally to monitor ketonuria, blood pressure and body measurements

**4.2 Exclusion Criteria**

- Any medical contraindication to use of VLCD, including lactation, presence of porphyria, severe hepatic disease, advanced renal disease, unstable cardiac disease (recent myocardial infarction or unstable angina), type 1 diabetes mellitus, overt psychosis, alcoholism or drug abuse^28^
- High alcohol intake based on NHMRC Australian Alcohol Guidelines^29^
- Patients in lithium, corticosteroids, anticonvulsants (although not a contraindication to Optifast, requires extra monitoring beyond the scope of this study)
- Evidence of extra-uterine disease clinically or on medically imaging
- Histological cell types including serous, clear cell, carcinosarcoma, sarcoma, de-differentiated
- Moderately differentiated or poorly differentiated (grades 2 and 3) endometrioid adenocarcinoma
- Inability to give informed consent
- Non English speaking
- European Cooperative Oncology Group (ECOG) performance status >3

**5 STUDY OUTLINE**

**5.1 Study flow chart**

Identification of motivated women with BMI ≥35 with a diagnosis of low risk endometrial cancer planned to undergo laparoscopic hysterectomy and meet inclusion/exclusion criteria requirements

🡻

Provision of written and verbal information regarding following a VLCD pre-operatively (including the Optifast Clinical Treatment Protocol booklet to discuss with their primary care giver/GP)

🡻

If interested, woman given patient information sheet and offered to participate in study

🡻

If woman agrees to study participation

Consent completed by treating gynaecologic oncologist

🡻

Enrolment into study and planned surgery date for 4-6 weeks

🡻

Nutritional assessment by dietician, Baseline fasting blood tests, anthropometric and blood pressure measurements

🡻

After 2 weeks of VLCD - contact with the dietitian/local GP to facilitate dietary adherence and success. Urinary ketones to assess dietary compliance. VLCD patient questionnaire.

Repeat anthropometric and BP measurements.

🡻

After 4 weeks of VLCD (if on 6 week program)– contact with dietitian/local GP, urinary ketones, VLCD patient questionnaire, anthropometric and BP measurements

🡻

On day of surgery (both 4 and 6 week patients)– repeat fasting blood tests, urinary ketones, VLCD patient questionnaire, anthropometric and BP measurements

🡻

Uterus, tubes, ovaries and any additional specimens sent to Australian Clinical Labs pathology for routine analysis

🡻

Data collection

🡻

Patient returns to normal diet and offered follow up with dietitian 1 month post operatively

**5.2 Scheduled Visits**

| Interventions | Enrolment Visit at initial consultation | Start VLCD | 2 weeks of VLCD | 4 weeks VLCD (if on 6 week program only) | On day of Surgery (both 4 and 6 week patients) |
| --- | --- | --- | --- | --- | --- |
| Informed Consent | ✓ |  |  |  |  |
| Inclusion/exclusion criteria, recording of history, physical exam | ✓ |  |  |  |  |
| Dietitian/ Anthropometric measures, BP |  | ✓ | ✓ | ✓ | ✓ |
| Fasting blood tests |  | ✓ |  |  | ✓ |
| Urine ketones |  |  | ✓ | ✓ | ✓ |
| Patient questionnaire re: VLCD |  |  | ✓ | ✓ | ✓ |
| Tissue collected for analysis |  |  |  |  | ✓ |

Not all of the above interventions are part of routine care for a patient diagnosed with an endometrial cancer. Whilst height, weight, BMI and BP are routine measurements at the initial consultation, anthropometric measures such as waist and hip circumference are not. All anthropometric measurements will be performed at baseline and fortnightly (on 2-3 occasions). Having a blood test (FBC, EUC, LFTs, CMP, coagulation profile, CA125) at baseline and on the day of surgery is routine (G&H) hence the additional tests particular to this research (uric acid, iron studies, fasting lipid profile -Total cholesterol, LDL, HDL, TG - fasting glucose metabolism -plasma glucose, plasma insulin, HBA1C -and 25OHVitD) required for the study can be performed at the time as the routine tests to avoid further invasive procedures. The patient will be required to provide a urine sample for urine ketone testing on 2-3 occasions. They will also be required to complete a 6 point likert questionnaire on 2-3 occasions. There will be fortnightly contact with the dietitian or local GP, either face-to-face or over the phone to facilitate dietary adherence and success.

**5.3 Study Procedure Risks**

Optifast VLCD is accepted as being safe with only minor transient side effects generally insufficient in duration or magnitude to warrant cessation. The main side effects of rapid weight loss and ketosis include cold insensitivity, halitosis, headache, alopecia, irritability, postural hypotension, fatigue, muscle cramps, bowel disturbance (constipation or diarrhoea).

Patients are still eligible for Optifast although extra care should be taken if there is a history of cholelithiasis, pancreatitis or gout as these conditions may flare during rapid weight loss.

Additionally the use of the VLCDs may influence the dosage requirements of some medications, however, patients on sulphonylureas, short and long acting insulins, diuretics for hypertension, medications for hyperlipidaemia, and warfarin are still eligible under medical supervision.

In Australia cancer surgery is normally assigned a clinical urgency Category 1 or 2 which means surgery is performed within 30-90 days of being added to a wait list (AIHW). The 4-6 week time frame will be at the discretion of the Gynaecologic Oncologist.

Venipuncture carries the small risk of swelling, tenderness, or inflammation at the site, vasovagal, infection and thrombosis.

**5.4 Recruitment and Screening**

Women recommended to have a VLCD as part of pre-operative preparation for surgery will be invited to participate. The consultant gynaecological oncologists accredited at SJOG Subiaco Hospital will identify them at their initial visit. A study coordinator will screen patients who are interested in participating. Patients’ medical history, current medications and BMI will be checked to ensure that all inclusion criteria are met and there are no exclusion criteria apparent. A patient information package and consent form will be given to the patient to read. The potential participant, if agreeable, will sign a written consent form for the study at the time of consenting to the laparoscopic surgical procedure. If they wish to have additional time to read the patient information package before making a decision to participate, a follow-up phone call addressing further questions and if they wish to participate will be made. It will be made clear to each participant that if they do not wish to participate or withdraw from the study, their ongoing care will not be affected.

**5.5 Informed Consent Process**

The patient will have adequate time to read the patient study information package and to ask any questions or concerns about the study. If they agree to participate, the treating team will gain their written consent at the time of the surgical consent. A follow-up phone call will be made to any potential participant who wishes to have further time to consider the study and consents to receive the call.

**5.6 Enrolment Procedure**

The participant will be enrolled into the study after the informed consent process has been completed and the participant has met all inclusion criteria and none of the exclusion criteria are apparent. The participant will receive a study enrolment number and this will be documented on the patient’s medical record and on all study documents.

**6 SAFETY**

**6.1 Study processes**

The collecting of blood will be by the usual processes ensuring sterility of patient/blood collector safety.

The transport of tissue samples from the operating theatre to Clinical Labs laboratory will be by the usual processes meeting all requirements for human tissue specimen collection.

**6.2 Data safety and monitoring**

## After the first 6 months of the study, the study coordinators will meet with a data monitoring committee to discuss progress and any safety issues relating to the study. If any issues are raised by patients and communicated with investigators in relation to the study, this will be communicated to DR Ayres immediately.

**6.3 National and international guidelines**

Approval for the study will be sought from the St John of God Healthcare Human Research Ethics Committee. Patients will be required to give informed written consent and will be free to withdraw from the study from any time during treatment.

This study will be conducted according to the National and International guidelines pertaining to ethical research on humans. The guidelines of the World Medical Association Declaration of Helsinki: Ethical Principles for Medical Research Involving Human subjects and the NHMRC National Statement on Ethical Conduct in Research involving Humans (2007), will be strictly adhered to at all times during the study.

**6.4 Early termination**

Early termination could occur in the event of poor recruitment, lack of funds, safety concerns or sickness in one of the principle investigators. The principle investigator, Dr Chloe Ayres would immediately inform HREC and the participants if the study were to be terminated early.

**7 STATISTICAL CONSIDERATIONS**

**7.1 Sample size and analysis plan**

The sample size for this observational, proof of concept study is pragmatic and analysis of will be descriptive. The findings of this study will facilitate calculation of the sample size calculation for a subsequent, appropriately powered, randomized controlled trial. Data analysis will be performed using the statistical software IBM SPSS Statistics Version 20.

**8 STORAGE AND ARCHIVING OF STUDY DOCUMENTS**

Clinical data for each patient will be given a code and de-identified from the patient details. The de-identified data will be kept in an Excel spreadsheet format on a computer, which can only be accessed by a login password unique to the investigators.

At the termination of the study, any paper copies of data will be shredded as per requirements for health related data. De-identified electronic files will be deleted two years after completion of the study as per the SJOG procedure for sensitive medical records. All data will be stored securely and destroyed at 7 years, as per NHMRC guidelines. The data will then be deposed of as per SJOG protocol for destruction of records.

**9 REFERENCES**

1. Australian Institute of Health and Welfare 2016
2. Oehler M. Robotics versus conventional laparoscopy for endometrial cancer: Where are we now? Maturitas 2015: 241-242.
3. MacKintosh ML, Crosbie EJ. Obesity-driven endometrial cancer: is weight loss the answer? BJOG 2013: 791-794
4. Crosbie EJ, Zwahlen M, Kitchener HC, Egger M, Renehan AG. Body mass index, hormone replacement theerapy and endometrial cancer risk: a meta-analysis. Cancer Epidemiol Biomarkers Prev 2010; 19(12): 3119-30.
5. Walls HL, Magliano DJ, Stevenson CE, Backholer K, Mannan HR, Shaw JE, and Peeters A (2012). Projected Progression of the Prevalence of Obesity in Australia. Obesity (Silver Spring). 20(4): 872-878.
6. Pavelka J, Ben-Shachar, Fowler J, Ramirez N, Copeland L, Eaton L, et al. Morbid obesity and endometrial cancer: surgical, clinical, and pathologic outcomes in surgically managed patients. Gynecol Oncol 2004; 95: 588-592.
7. El Safadi S, Sauerbier A, Hackethal A, Munsteadt K. Body weight changes after the diagnosis of endometrial cancer and their influences on disease-related prognosis. Arch Gynecol Obstet 2012; 285: 1725-1729
8. McCourt C, Mutch D, Gibb R, Rader J, Goodfellow P, Trinkaus K et al. Body mass index: Relationship to clinical, pathologic and features of microsatellite instability in endometrial cancer 2007; 104: 535-539.
9. [Yu CK](http://www.ncbi.nlm.nih.gov/pubmed/?term=Yu%20CK%5BAuthor%5D&cauthor=true&cauthor_uid=15663410), [Cutner A](http://www.ncbi.nlm.nih.gov/pubmed/?term=Cutner%20A%5BAuthor%5D&cauthor=true&cauthor_uid=15663410), [Mould T](http://www.ncbi.nlm.nih.gov/pubmed/?term=Mould%20T%5BAuthor%5D&cauthor=true&cauthor_uid=15663410), [Olaitan A](http://www.ncbi.nlm.nih.gov/pubmed/?term=Olaitan%20A%5BAuthor%5D&cauthor=true&cauthor_uid=15663410).Total laparoscopic hysterectomy as a primary surgical treatment for endometrial cancer in morbidly obese women. BJOG 2005; 112(1):115-7.
10. O’Gorman T, MacDonald N, Mould T, Cutner A, Hurley R, Olaitan A. Total laparoscopic hysterectomy in morbidly obese women with endometrial cancer anaesthetic and surgical complications. Eur J Gynaec Oncol 2008: 171-173.
11. Walter J, Piedmonte M, Spirtos N, Eisenkop S, Schlaerth J, Mannel R. Laparoscopy Compared with Laparotomy for Comprehensive Surgical Staging of Uterine Cancer: Gynecologic Oncology Group Study LAP2. J Clin Oncol 2009; 27 (32): 5331-5336.
12. Scribner D, Waler J, Johnson G, McMeekin, Gold M, Mannel R. Gynecol Oncol 2002; 84: 426-430.
13. Pellegrino A, Signorelli M, Fruscio R, Villa A, Buda A, Beretta P, et al. [Feasibility and morbidity of total laparoscopic radical hysterectomy with or without pelvic limphadenectomy in obese women with stage I endometrial cancer.](http://wiki.cancer.org.au/australia/Citation:Pellegrino_A,_Signorelli_M,_Fruscio_R,_Villa_A,_Buda_A,_Beretta_P,_et_al_2009) Arch Gynecol Obstet 2009 May;279(5):655-60
14. Childers J, Hatch K, Tran A, Surwit E. Laparoscopic paraaortic lymphadenectomy in gynecologic malignancies. Obstet Gynecol 1993; 82: 741-7.
15. Manolitsas T, McCartney A. Total Laparoscopic Hysterectomy in the Management of Endometrial Carcinoma. J Am Assoc Gynecol Laparosc 2002: 9(1): 54-62.
16. Obermair A, Manolitsas TP, Leung Y, Hammond IG, McCartney AJ. [Total laparoscopic hysterectomy versus total abdominal hysterectomy for obese women with endometrial cancer.](http://wiki.cancer.org.au/australia/Citation:Obermair_A,_Manolitsas_TP,_Leung_Y,_Hammond_IG,_McCartney_AJ_2005) Int J Gynecol Cancer 2005;15(2):319-24
17. O'Gorman T, MacDonald N, Mould T, Cutner A, Hurley R, Olaitan A. [Total laparoscopic hysterectomy in morbidly obese women with endometrial cancer anaesthetic and surgical complications.](http://wiki.cancer.org.au/australia/Citation:O%27Gorman_T,_MacDonald_N,_Mould_T,_Cutner_A,_Hurley_R,_Olaitan_A_2009) Eur J Gynaecol Oncol 2009;30(2):171-3.
18. Kadar N. Laparoscopic pelvic lymphadenectomy in obese women with gynecologic malignancies. J Am Assoc Gynecol Lap 1995; 2: 163-7.
19. Eltabbakh G, Shamonki M, Moody J, Garafano L. Hysterectomy for obese women with endometrial cancer: laparoscopy or laparotomy? Gynecologic Oncology 2000; 78: 329-335
20. Holub Z, Bartos P, Jabor A, Eim J, Pischlova D, Kliment L. et al 2000. J Am Assoc Gynecol Laparosc; 7(1): 83-88
21. Janda M, Gebski V, Forder P et al. Total laparoscopic versus open surgery for stage 1 endometrial cancer: the LACE randomized controlled trial. Contemp Clin Trials 2006; 27(4): 353-63.
22. Colles S, Dixon J, Marks P, Strauss B, O’Brien P. Preoperative weight loss and a very-low-energy diet: quantitation of changes in liver and abdominal fat by serial imaging. Am J Clin Nutr 2006; 84: 304-11.
23. Mustajoki P, Pekkarinen T. Very low energy diets in the treatment of obesity. The International Association for the Study of Obesity 2001: 61-72
24. Leonetti F, Campanile F, Coccia, Capoccia D, Alessandroni L, Puzziello A et al. Very Low-Carbohydrate Ketogenic Diet before Bariatric Surgery: Prospective Evaluation of a Sequential Diet. Obes Surg 2015; 25: 64-71.
25. Pekkarinen T, Mustajoki P. Use of Very Low- Calorie Diet in Preoperative Weight Loss: Efficacy and Safety. Obesity Research 1997; 5(6) 595-602.
26. Van Nieuwenhove Y, Dambrauskas Z, Campillo-Soto A, Van Dielen F, Wiezer R, Janssen I, Kramer M, Thorell A. Preoperative Very Low-Calorie Diet and Operative Outcome After Laparoscopic Gastric Bypass – A Radomized Multicenter Study. Arch Surg 2011; 146(11): 1300-1305.
27. Jones A, Waterland P, Powell-Brett S, Super B, Richardson M, Bowley D. Preoperative Very Low-Calorie Diet Reduces Technical Difficulty During Laparoscpic Cholecystectomy in Obese Patients. Surg Laparosc Endosc Percutan Tech 2016; 26(3): 226-229.
28. Very low-calorie diets. National Task Force on the Prevention and Treatment of Obesity, National Institues of Health. JAMA 1993; 270: 967-74.
29. Australian Alcohol guidelines. Health risks and benefits. 2001, National Health and Medical Research Council. Internet: http://www.nhmrc.gov.au/publications/_files/ds.pdf
30. Henry RR, Wiest-Kent TA, Scheaffer L, Kolterman OG, Olefsky JM. Metabolic consequences of very-low-calorie diet therapy in obese non-insulin –dependent diabetic and nondiabetic subjects. Diabetes1986; 35: 155-64.
31. Lewis M, Phillips M, Slavoinek J, Kow L, Thompson C, Toouli J. Change in liver size and fat content after treatment with optifast very low calorie diet. Obesity Surgery 2006; 16: 697-701.
32. Argenta PA, Kassing M, Truskinovsky AM, Svendsen CA. Batiatric Surgery and endometrial pathology in asymptomatic morbidly obese women: a prospective, pilot study. BJOG 2013; 120; 795-800.
33. Gallos ID, Yap J, Rajkhowa M, Luesley DM, Coomarasamy A, Gupta J. Regression, relapse, and live birth rates with fertility-sparing therapy for endometrial cancer and atypical complex endometrial hyperplasia: a systematic review and metaanalysis. AJOG 2012; 207;266.e1-12.
34. Siedhoff MT, Clark LH, Hobbs KA, Findley AD, Moulder JK, Garrett JM. Mechanical bowel preparation before laparoscopic hysterectomy. A randomized controlled trial. Obstet Gynecol. 2014;123:562–567.
35. [Won H](http://www.ncbi.nlm.nih.gov/pubmed?term=Won%20H%5BAuthor%5D&cauthor=true&cauthor_uid=23635616), [Maley P](http://www.ncbi.nlm.nih.gov/pubmed?term=Maley%20P%5BAuthor%5D&cauthor=true&cauthor_uid=23635616), [Salim S](http://www.ncbi.nlm.nih.gov/pubmed?term=Salim%20S%5BAuthor%5D&cauthor=true&cauthor_uid=23635616), [Rao A](http://www.ncbi.nlm.nih.gov/pubmed?term=Rao%20A%5BAuthor%5D&cauthor=true&cauthor_uid=23635616), [Campbell NT](http://www.ncbi.nlm.nih.gov/pubmed?term=Campbell%20NT%5BAuthor%5D&cauthor=true&cauthor_uid=23635616), [Abbott JA](http://www.ncbi.nlm.nih.gov/pubmed?term=Abbott%20JA%5BAuthor%5D&cauthor=true&cauthor_uid=23635616). Surgical and patient outcomes using mechanical bowel preparation before laparoscopic gynecologic surgery: a randomized controlled trial. Obstet Gynecol. 2013;121:538–546.

**10 APPENDICES**

**10.1 Initial visit history form.**

**GYNAECOLOGICAL ONCOLOGY**

**PATIENT INFORMATION**

**HISTORY OF PRESENT ILLNESS: Date:**

**BLADDER:**

**BOWELS:**

**A WEIGHT LOSS B Yes, how much (kg)**

No = 0 1-05 - 5kg = 1

Yes = 2 6 – 10kg = 2

Unsure = 2 11 – 15kg = 3

> 15kg = 4

Unsure = 5

**C. EATING POORLY OR LACK OF NUTRITION SCORE**

**APPETITE** If > 2 refer for nutrition

assessment/intervention

No = 0

Yes = 1 **Total score =**

**OBSTETRIC HISTORY:**

**MENSTRUAL HISTORY:**

**Cycle:**

**Menarche:**

**Menopause:**

**Years on OC’s:**

**HRT:**

**ANY PREVIOUS FERTILITY DRUGS:**

**SEXUAL HISTORY:**

**PAP SMEAR HISTORY:**

**MAMMOGRAM:**

**PAST MEDICAL HISTORY:**

**MEDICATIONS**

**PAST SURGICAL HISTORY**

**PREVIOUS CHEMOTHERAPY:**

**PREVIOUS RADIOTHERAPY:**

**CIGARETTES:**

**ALCOHOL:**

**ALLERGIES:**

**FAMILY HISTORY OF SERIOUS ILLNESS/CANCER:**

**SOCIAL HISTORY**

**PHYSICAL EXAMINATION:**

**General Appearance:**

**Performance Status:**

**Height: Weight: BMI:**

**BP:**

**Chest:**

**Heart:**

**Breasts:**

**Abdomen:**

**Peripheral Nodes:**

**PELVIC EXAMINATION:**

**External Genitalia:**

**Vagina:**

**Cervix:**

**Uterus:**

**Adnexae:**

**Rectal:**

**COLPOSCOPY:**

**PROVISIONAL DIAGNOSIS:**

**INVESTIGATIONS:**

**TREATMENT:**

**10.2 Patient VLCD Questionnaire**

**Circle how many weeks of Optifast have you completed?**

| 2 | 4 | 6 |
| --- | --- | --- |

1. **How do you rate the product taste?**

| Highly unacceptable | Unacceptable | Tolerable | Acceptable | Highly acceptable |
| --- | --- | --- | --- | --- |
| 1 | 2 | 3 | 4 | 5 |

1. **How hungry are you?**

| Extreme Hunger | Hungry most days | Hungry some of the time | Occasional Hunger | No Hunger |
| --- | --- | --- | --- | --- |
| 1 | 2 | 3 | 4 | 5 |

1. **How much nausea or vomiting do you have?**

| Daily | 4-6 times/week | 2-3 times/week | ≤1 time/week | None |
| --- | --- | --- | --- | --- |
| 1 | 2 | 3 | 4 | 5 |

1. **How well are your bowels working compared to what is normal for you?**

| no bowel motion in the past 4 days | no bowel movement in the past 2-3 days | normal | increased frequency | diarrhoea |
| --- | --- | --- | --- | --- |
| 1 | 2 | 3 | 4 | 5 |

1. **How often are you emotional eating?** - Emotional eating defined as ‘eating foods outside the diet’s guidelines, due to emotional reasons such as stress, sadness, frustration, anger.

| Daily | 4-6 times/ week | 2-3 times/week | ≤ 1 time/week | None |
| --- | --- | --- | --- | --- |
| 1 | 2 | 3 | 4 | 5 |

1. **How often are you social eating?** - Social eating defined as eating foods outside the diet’s guidelines because you are in a social setting.

| Daily | 4-6 times/ week | 2-3 times/week | ≤ 1 time/week | None |
| --- | --- | --- | --- | --- |
| 1 | 2 | 3 | 4 | 5 |

**Funding**

- $12,500 research grant from Australian Society of Gynaecologic Oncologists (ASGO)
